# Obesity and Cardiometabolic Risk Factors in Children and Young Adults With Non-classical 21-Hydroxylase Deficiency

**DOI:** 10.3389/fendo.2019.00698

**Published:** 2019-10-11

**Authors:** Liat de Vries, Yael Lebenthal, Moshe Phillip, Shlomit Shalitin, Ariel Tenenbaum, Rachel Bello

**Affiliations:** ^1^The Jesse Z and Sara Lea Shafer Institute for Endocrinology and Diabetes, National Center for Childhood Diabetes, Schneider Children's Medical Center of Israel, Petah Tikva, Israel; ^2^Sackler Faculty of Medicine, Tel Aviv University, Tel Aviv-Yafo, Israel

**Keywords:** cardiometabolic syndrome, children and adolescents, non-classical congenital adrenal hyperplasia, overweight, obesity

## Abstract

**Introduction:** Classical congenital adrenal hyperplasia is associated with an increased risk of obesity and cardiometabolic disease. The aim of the study was to determine if this is also true for non-classical congenital adrenal hyperplasia (NCCAH).

**Methods:** A retrospective, cross-sectional, single-center study design was used. Data were collected on 114 patients (92 female) with NCCAH diagnosed during childhood/adolescence at a tertiary medical center. Patients were classified by treatment status at the last clinic visit. Outcome measures were assessed at diagnosis and the last clinic visit: weight status, body composition, blood pressure, lipid profile, and glucose metabolism. The prevalence of overweight/obesity was compared to the parental prevalence, and for patients aged 11–20 years, to the Israeli National Survey.

**Results:** Mean age was 7.9 ± 4.2 years at diagnosis and 17.1 ± 6.9 years at the last follow-up. At the last clinic visit, 76 patients were under treatment with glucocorticoids, 27 were off-treatment (previously treated), and 11 had never been treated. The rate of obesity (11.4%) was similar to the parental rates, and the rate of overweight was significantly lower. In patients 11–20 years old, rates of obesity or obesity + overweight were similar to the general Israeli population (11.4 vs. 15.1%, *P* = 0.24 and 34.2 vs. 41.6% *P* = 0.18, respectively). No significant difference was found between glucocorticoid-treated and off-treatment patients in any of the metabolic or anthropometric parameters evaluated, except for a lower mean fat mass (% of body weight) in off-treatment patients (23.0 ± 7.7% vs. 27.8 ± 6.8%, *P* = 0.06). Systolic hypertension was found in 12.2% of NCCAH patients either treated or untreated.

**Conclusion**: NCCAH diagnosed in childhood, whether treated or untreated, does not pose an increased risk of overweight, obesity, or metabolic derangements in adolescence and early adulthood.

## Introduction

Non-classical congenital adrenal hyperplasia (NCCAH) due to 21-hydroxylase deficiency is an autosomal recessive disease of the adrenal cortex caused by mutations in the *CYP21A2* gene. The disease frequency is estimated at 0.1% of the general population, but it is significantly higher in some ethnic groups: 1–2% in Hispanics and Yugoslavians and 3–4% in Ashkenazi (Eastern European) Jews (1, 2).

In childhood, NCCAH may manifest with premature pubarche, signs of hyperandrogenism such as acne, hirsutism, and irregular menses (2–5), and advanced bone age which may lead to short adult height (6, 7). Treatment consists of glucocorticoids in doses aimed at suppressing hyperandrogenism, which are usually higher than the physiologic replacement and cannot mimic the physiological circadian rhythm of cortisol. Given the controversial findings in the literature regarding the need for treatment of NCCAH (2, 8), clinicians need to weigh the benefits against the risks of steroid-induced hyperglycemia, weight gain, increased blood pressure, and hyperlipidemia (9). The presence of three or more of these conditions leads to the metabolic syndrome, increasing the risk of cardiovascular morbidity and mortality. At the same time, chronic androgen excess may contribute to the metabolic disorder by its association with increased visceral adiposity and insulin resistance and their metabolic consequences (10, 11). Adolescents and adults with the classical form of congenital adrenal hyperplasia (CAH) appear to have an increased risk of obesity and cardiometabolic risk factors (12–14). Studies on cardiometabolic risk factors in pediatric and young adult patients with the non-classical form are scarce (15, 16).

The aim of the present study was to investigate the prevalence of overweight and obesity among adolescents and young adults with NCCAH and to assess metabolic risk factors in these patients as well as the association of these risk factors with treatment.

## Patients and Methods

### Study Population

A retrospective, cross-sectional study design was used. The cohort consisted of 114 consecutive unselected patients with NCCAH (92 female) attending a tertiary pediatric endocrinology institute from 1986 to 2017. Inclusion criteria were diagnosis before 18 years of age and diagnosis on the basis of an ACTH stimulated 17-hydroxyprogesterone (17-OHP) serum level of ≥45 nmol/L (*n* = 100, molecular confirmation in 67) or a level of >30 nmol/l if confirmed by molecular analysis.

### Treatment

Therapy with glucocorticoids was initiated in symptomatic patients, namely children with early onset, and rapid progression of pubarche or bone age advancement and adolescent girls with overt virilization. Doses were titrated according to growth and clinical parameters as well as the hormonal profile in order to maintain androstenedione levels at sex- and age-appropriate levels and 17-OHP levels at <30 nmol/l. Glucocorticoid dosage was expressed as hydrocortisone per square meter of body surface. When glucocorticoid treatment was administered in the form of prednisolone rather than hydrocortisone, the total dose of prednisolone was multiplied by 4 to yield an equivalent dose of hydrocortisone.

### Clinical Methodology

Clinical data were extracted from the medical files as follows: age at diagnosis, reason for referral, height, weight, secondary sex characteristics, and laboratory results. Outcome measures were assessed at diagnosis and the last clinic visit: weight status, body composition, blood pressure, lipid profile, and glucose metabolism.

Pubertal stage was classified according to Tanner and Marshall (17, 18) at diagnosis, as follows: prepubertal (Tanner 1), active puberty (Tanner 2–4), and fully pubertal (Tanner 5). Body weight and height were measured to the nearest 0.1 kg and 0.1 cm, respectively, using a Harpenden stadiometer (Holtain Ltd, Crosswell, UK), and a balanced scale. Body mass index (BMI) was calculated. The height and weight of both parents were measured when possible as part of the clinic routine. The height, weight, and BMI-standard deviation score (SDS) were calculated and assessed according to the recommendations of the Centers for Disease Control and Prevention (19). In patients younger than 18 years, overweight was defined as a BMI within the 85–94th percentile for children of the same age, and obesity as a BMI ≥the 95th percentile. In older patients, overweight was defined as BMI 25–29.99, and obesity as BMI ≥30 (20). The prevalence of overweight and obesity at the last visit was calculated for the entire cohort. Data of patients in grades 11–20 years old (*n* = 76) were compared to the National Health and Nutrition Survey (21).

Systolic blood pressure (SBP) and diastolic blood pressure (DBP) were measured in all subjects using the standardized automated Datascope Accutorr Plus (Soma Technology, Bloomfield, USA) on the right arm. SBP and DBP percentiles adjusted for sex, age, and height were calculated using the American Academy of Pediatrics guidelines (22). Patients were considered hypertensive if the average SBP and/or DBP was at or above the 95th percentile for sex, age, and height on three or more occasions.

Bone age was evaluated according to Greulich and Pyle (23), with the delta of bone age minus chronological age calculated accordingly.

The cohort was subdivided according to treatment status at the last visit: currently treated (*n* = 76), off-treatment (previously treated, *n* = 27), and untreated (never treated, *n* = 11), and again by gender.

### Biochemical Analysis

The hormonal evaluation included 17-OHP and cortisol levels at presentation, baseline, and following intravenous administration of 0.25 mg Synacthen (Novartis, New York, NY, USA). In post-menarcheal girls, the test was conducted in the early follicular phase of the menstrual cycle. Follow-up basal androgen levels were measured every 6 months, or earlier when dose adjustment was required. All hormonal analyses were performed in the endocrine laboratory of our hospital with commercial kits; serum cortisol and 17-OHP were measured using the Coat-A-Count radioimmunoassay (Diagnostics Products Corporation, Los Angeles, CA, USA).

Annual fasting measurements of glucose, lipid profile, and liver function tests were performed as part of the routine standard of care. Patients were instructed to take the medication after blood samples were collected. Serum glucose was measured with the glucose oxidase colorimetric method (Hitachi 917 automated analyzer, Roche Diagnostics, Mannheim, Germany), and serum total cholesterol, triglycerides, and high-density lipoprotein (HDL)-cholesterol were measured with the enzymatic colorimetric method (Hitachi 904 automated analyzer, Roche Diagnostics). Low-density lipoprotein (LDL) was calculated according to the Friedewald formula: LDL, total cholesterol (TC)— HDL—triglycerides (TG)/5. The most recent data available were used for analysis. Fasting plasma TG concentrations of >110 mg/dl were considered elevated, and HDL <40 mg/dl was considered low (24). Additionally, the percentiles of HDL, LDL, TG, and age- and sex-adjusted TC were calculated relative to published normal values (25).

### Extensive Metabolic Evaluation

A subgroup of 38 patients from the cohort underwent further assessment. The patients were consecutively and prospectively recruited during a routine visit to the clinic over 1 year and signed an informed consent form. The evaluation included measurement of waist and hip circumference, skin-fold thickness (iliac, scapular, and triceps), and body composition using a bioelectrical impedance analyzer (Tanita SC-331S, Tokyo, Japan). Measured values of fat mass were compared to the accepted normal range for age and sex (26, 27). Waist circumference was measured at the minimum circumference between the iliac crest and the rib cage, and hip circumference was measured at the maximum protuberance of the buttocks. The waist-to-hip ratio was calculated (28). Waist circumference percentiles were determined according to the estimated value for percentile regression for European and American children and adolescents (29). For female participants aged 11–20 years, mean and median waist and hip circumference, in addition to waist-to-hip ratio (as too few such measurements were available for male patients), were compared to the National Health and Nutrition Survey values for the same age group (21).

Insulin resistance (IR) was estimated using the homeostasis model assessment (HOMA) method (IR = insulin [μmol/mL] × glucose [mmol/L]/22.5) (30).

The consensus definition of the International Diabetes Federation (IDF) was used for the diagnosis of metabolic syndrome in children and adolescents. Patients were diagnosed with metabolic syndrome if they met at least 3 of the 5 criteria listed by the consensus (29).

The study was approved by the Institutional Review Board of Rabin Medical Center, Israel.

### Statistical Analysis

Analyses were carried out using BMDP statistical software (University of California Press, Los Angeles, CA, USA) (31). Data were expressed as means and standard deviations for normally distributed variables and medians and interquartile ranges for variables with a skewed distribution. For continuous variables, groups were compared using analysis of variance. Discrete variables were compared using Pearson's chi-square test or Fisher's exact test, as appropriate. For variables that did not have a Gaussian distribution, and because of the extremely small numbers, the Mann–Whitney *U*-test was used to compare groups. A *p* value of ≤0.05 was considered significant.

## Results

The baseline characteristics of the study cohort are detailed in Table 1. Mean patient age at diagnosis was 7.9 ± 4.2 years (range, 5 months-18 years), 7.1 ± 4.4 years in girls and 8.4 ± 4.0 years in boys, and at the last follow-up, 17.1 ± 6.9 years. The mean duration of follow-up was 9.2 ± 6.6 years (median 8.25 years). At diagnosis, 54 patients (65% females) were prepubertal, 48 (92% females) were in active puberty, and 12 (all females) were fully pubertal. The main reason for referral, in 56 patients, was premature pubarche, either isolated or associated with signs of gonadarche. The remaining 58 patients were referred for hyperandrogenism in adolescence (*n* = 16), a family history of NCCAH (*n* = 29), clitoromegaly (*n* = 5), or short stature associated with advanced bone age (*n* = 8).

**Table 1:** Clinical characteristics at diagnosis of 114 children and adolescents with NCCAH.

**Characteristics**	**All (*n* = 114)**	**Currently treated (*n* = 76)**	**Off-treatment (*n* = 27)**	**Untreated (*n* = 11)**
Female: male, *n*	92:22	63:13	20:7	8:3
Age at diagnosis (years)	7.9 ± 4.2 (0.4–17.9)	8.2 ± 4.0 (0.7–17.9)	7.5 ± 3.8 (0.4–17.0)	7.8 ± 5.7 (1.2–17.5)
Age at treatment initiation (years)	8.6 ± 3.4	8.5± 3.3	9.1 ± 3.6	
1st year hydrocortisone^*^ dose (mg/m^2^)	10.6 ± 4.9	10.7 ± 4.9	10.2 ± 3.8	
Height-SDS	0.10 ± 1.22	0.14 ± 0.13	0.35 ± 0.89	−1.07 ± 1.62^†^
Weight-SDS	0.22 ± 1.23	0.27 ± 0.18	0.52 ± 0.83	−0.99 ± 1.93
BMI-SDS	0.35 ± 1.13	0.33 ± 1.09	0.62 ± 0.87	−0.21 ± 1.77
Δ Bone age- chronological age (years)	1.06 ± 1.26	1.29 ± 1.24	0.88 ± 1.29	0.00 ± 0.83^§^
Basal 17-OHP (nmol/l)	28.7 ± 27.6 (2.4–174)	33.8 ± 29.9^‡^ (3.2–174)	23.2 ± 23.7^‡^ (2.7–93.8)	10.5 ± 7.1^§^ (3.2–23.7)
Peak 17-OHP (nmol/l)	129.5 ± 96.8	143.7 ± 106.8^‡^^*^	105.6 ± 74.9^‡^	88.2 ± 52.4
Stimulated cortisol level (nmol/l)	567 ± 165	536 ± 132	641 ± 148	609 ± 172
Androstenedione (nmol/l)	4.1 ± 3.9	4.9 ± 4.4^‡^	3.0 ± 2.1^‡^	1.0 ± 0.7^§^
Paternal BMI	26.7 ± 3.9	26.8 ± 3.8	26.9 ± 4.5	26.2 ± 3.4
Paternal BMI-SDS	0.93 ± 0.95	0.95 ± 0.98	0.95 ± 0.97	0.87 ± 0.87
Maternal BMI	26.0 ± 5.1	25.8 ± 4.9	26.8 ± 5.6	23.1 ± 4.2
Maternal BMI-SDS	0.76 ± 0.86	0.75 ± 0.86	0.91 ± 0.81	0.16 ± 1.04

*Data are shown as mean ± SD (range) but analyzed using the non-parametric Mann-Whitney U-test since the sample sizes are very small*.

* *If prednisolone was administered, the total dose of prednisolone was multiplied by 4 to yield an equivalent dose of hydrocortisone*.

† *P < 0.05 for untreated vs. off-treatment patients*.

‡ *P < 0.05 for treated vs. off-treatment*.

§ *P < 0.01 for untreated vs. currently treated*.

*17-OHP, 17-hydroxyprogesterone; BMI, body mass index; NCCAH, non-classical congenital adrenal hyperplasia; SDS, standard deviation score*.

Glucocorticoid treatment was administered when clinically indicated. Treatment was initiated at a mean age of 8.6 ± 3.4 years; 81% of the cohort started treatment before age 10 years. At the last clinic visit, 76 patients were being treated with glucocorticoids (daily hydrocortisone or hydrocortisone-equivalent dose was 9.2 ± 4.5 mg/m^2^), 27 had terminated treatment at a mean of 4.1 ± 0.9 years previously, and 11 had never been treated. The mean duration of steroid treatment was 7.3 ± 6.3 years for the glucocorticoid-treated group and 6.5 ± 5.2 years for the off-treatment group (*p* = 0.5).

Of the 11 patients who were never treated, 4 were diagnosed incidentally when evaluated for short stature, 4 were evaluated because of a family history of NCCAH, and 3 presented with mild premature pubarche without bone age advancement. This subgroup differed clinically from the rest of the cohort (Appendix I in Supplementary Material).

### Overweight and Obesity

At the last clinic visit, the rate of overweight for the entire cohort was 21.9%, and of obesity, 11.4%, with no significant difference between patients who were older or younger than 20 years (Table 2). Rates of overweight and obesity for patients who were 11–20 years old at the last visit were comparable to the rates reported for the same age group in the general population. Rates of overweight were significantly lower in the patients than in their mothers (*p* = 0.01) and fathers (*p* < 0.001). The duration of steroid treatment was inversely associated with BMI-SDS (*r* = −0.20, *P* < 0.05).

**Table 2:** Prevalence of overweight and obesity at last follow-up in children/adolescents with NCCAH by age at last visit (< or>20) compared to the Israel National Survey for ages 11–20 years and compared to mothers and fathers.

**Parameter**	***N***	**Overweight (%)**	**Obese (%)**
All patients	114	21.9	11.4
**Comparison by age at last visit**			
<20 years	76	23.6	10.5
>20 years	38	18.4	13.2
*P* value		0.3	0.4
**Comparison with national survey (for age 11–20 years)**			
Age 11–20 years, study	76	23.6	10.5
Age 11–20 years, national survey	3,443	26.5	15.1
*P* value		0.24	0.18
**Comparison with parental values**			
Mothers	100	36.8	13.6
*P* value		0.01	0.4
Fathers	86	52.5	14.5
*P* value		<0.001	0.3

*NCCAH, non-classical congenital adrenal hyperplasia*.

### Cardiometabolic Risk Factors

None of the patients was classified as having the metabolic syndrome according to either the criteria of Ford (24) or the IDF consensus (27).

Systolic hypertension (SBP >95th percentile) was found in 12.2% of the cohort: in 10.5% of the currently treated group, in 18.5 % of the off-treatment group and in 9% of the untreated group (*p* = 0.3). SBP and SBP percentile were associated with BMI-SDS (*r* = 0.275, *p* = 0.009, and *r* = 0.28, *p* < 0.02, respectively). Among the 17 patients with systolic hypertension, only 4 had a BMI-SDS consistent with overweight or obesity. DBP above the 95th percentile was measured in 2.2% of the cohort: in 3.5% of the treated group and in none of the off-treatment group (*p* = 0.49). No association was found between DBP and BMI-SDS.

In a search for the explanation for the high rate of patients with systolic hypertension, we investigated a possible association of SBP and SBP percentile with the following variables: birthweight and levels of serum androstenedione, testosterone, and 17-OHP at diagnosis and at last visit. No association was found for any of these variables. This held true for DBP and DBP percentile as well.

Steroid treatment duration was associated with SBP (*r* = 0.26, *P* < 0.05) and DBP (*r* = 0.31, *P* < 0.01), but not with sex-, age-, and height-adjusted blood pressure percentiles (for SBP%: *r* = 0.09, *P* = NS; for SDP%: *r* = 0.15, *p* = NS).

There was no significant association between treatment duration and levels of total and LDL-cholesterol, TG, fasting glucose, HOMA-IR, skin-fold thickness, and body fat mass (as percentiles). The dose at the last clinic visit was associated only with higher LDL-cholesterol (*r* = 0.46, *P* < 0.05).

Waist circumference was negatively correlated with HDL (−0.49, *P* < 0.02) but not with LDL, total cholesterol, or TG level. In the female adolescent patients, waist circumference and waist/hip ratio were similar to values in the national survey: for girls 11–20 years old mean waist circumference was 73.5 cm (median 73.8) compared to 73.1 cm (median 73.8 cm) in the national survey (*P* = 0.25), and mean waist-to-hip ratio was 0.78 (median 0.79) compared to 0.77 (median 0.76) in the national survey (*P* = 0.6). Hip circumference was associated with glucocorticoid therapy duration (*r* = 0.542, *p* < 0.005) and current dosage (*r* = 0.478, *p* < 0.05). On stepwise regression analysis, only BMI-SDS and age were strongly associated with hip circumference (multiple *R*^2^ = 0.88).

Effects of dose and duration of therapy are presented in Table 3.

**Table 3:** Effects of glucocorticoid therapy duration and dose on cardiometabolic risk factors in patients with NCCAH, currently-treated and off- treatment (*N* = 103).

**Risk factors**	**Therapy duration**		**1st year dose**		**Current dose**	
	***r***	***P***	***r***	***P***	***r***	***P***
Current BMI-SDS	−0.202	<0.05	0.074	ns	0.033	ns
Treatment duration			0.359	<0.001	0.106	ns
SBP (mmHg)	0.262	<0.05	0.226	<0.05	−0.126	ns
SBP percentile	0.089	ns	0.241	<0.05	−0.14	ns
DBP (mmHg)	0.309	<0.01	0.29	<0.02	0.007	ns
DBP percentile	0.15	ns	0.257	<0.05	−0.07	ns
% fat by bioelectrical impedance	0.075	ns	0.200	ns	0.046	ns
Iliac skinfold (mm)	0.306	ns	0.15	ns	0.22	ns
Triceps skinfold (mm)	0.419	ns	0.17	ns	0.12	ns
Scapular skinfold (mm)	0.267	ns	0.12	ns	0.30	ns
Waist circumference (cm)	0.264	ns	−0.08	ns	0.33	ns
Hip circumference (cm)	0.542	<0.005	−0.21	<0.005	0.478	<0.05
Waist/hip ratio	−0.507	<0.005	0.295	<0.01	−0.37	ns
Total cholesterol	0.09	ns	−0.186	ns	0.289	ns
HDL	0.22	<0.01	−0.057	ns	0.009	ns
LDL	0.159	ns	−0.250	ns	0.46	<0.05
Triglycerides	−0.083	ns	−0.43	ns	−0.08	ns
Glucose	0.244	ns	0.168	ns	0.125	ns
Insulin	−0.32	ns	0.36	ns	−0.53	ns

*DBP, diastolic blood pressure; HDL, high-density lipoprotein; LDL, low-density lipoprotein; NCCAH, nonclassical congenital adrenal hyperplasia; ns, not significant; SBP, systolic blood pressure*.

### Analysis by Glucocorticoid Treatment Status

There were no differences in clinical characteristics between the patients receiving treatment or those off treatment at the last follow-up visit (Table 4).

**Table 4:** Clinical characteristics by treatment status at last follow-up in patients with NCCAH.

**Characteristics**	**Currently treated** **(*N* = 76)**	**Off-treatment** **(*N* = 27)**	**Untreated** **(*N* = 11)**
Age (years)	17.4 ± 7.1	18.7 ± 5.7	11.4 ± 5.7^*^
Tanner (1, 2–4, 5)	8, 14, 54	3, 1, 23	3, 5, 3
Follow-up duration	9.1 ± 6.5	11.2 ± 6.9	3.6 ± 2.6^†^
Treatment duration (years)	7.3 ± 6.3	6.5± 5.2	
Weight-SDS	0.39 ± 0.99	0.37 ± 0.92	−0.69 ± 1.75
Height-SDS	−0.44 ± 0.99	−0.36 ± 0.83	−0.57 ± 1.76
BMI-SDS	0.65 ± 0.89	0.64 ± 0.73	−0.37 ± 1.23^*^
SBP (mmHg)	113.3 ± 10.9	118.1 ± 9.3	108.2 ± 14.5
SBP percentile	61.2 ± 27.1	75.1 ± 18.0	48.7 ± 33.1
DBP (mmHg)	66.7 ± 9.5	69.8 ± 7.2	61.1 ± 6.9
DBP percentile	56.1 ± 26	66.2 ± 19.7	41.0 ± 21.8
17OHP (nmol/l)	22.0 ± 29.5	23.9 ± 19.1	10.2 ± 9.5
Androstenedione (nmol/l)	6.4 ± 4.1^‡^	12.1 ± 8.4^‡^	3.5 ± 4.5
Testosterone (nmol/l)	2.5 ± 4.8	2.5 ± 3.5	0.2 ± 0.5^*^

*Data are mean ±SD unless otherwise specified*.

* *P < 0.05 for untreated vs. off-treatment and currently treated groups*.

† *P < 0.01 for untreated vs. off-treatment and currently treated groups*.

‡ *P < 0.05 for treated vs. off-treatment*.

*17-OHP, 17-hydroxyprogesterone; BMI, body mass index; DBP, diastolic blood pressure; NCCAH, nonclassical congenital adrenal hyperplasia; SBP, systolic blood pressure; SDS, standard deviation score*.

At the last clinic visit, the group receiving treatment had a tendency to higher mean fat mass than the untreated group (27.8 ± 6.8 vs. 23.0 ± 7.7% of body weight, *p* = 0.06) and a higher percentage of patients with fat mass (expressed in percent) above the accepted normal range for age and sex (38.8 vs. 7.1%, *p* < 0.05). They also had a tendency for higher weight-SDS and BMI-SDS. No significant differences were found between the treated and off-treatment patients in lipid profile, levels of glucose and insulin, or HOMA-IR (Table 5).

**Table 5:** Cardiometabolic risk factors by treatment status in patients with NCCAH who underwent extensive evaluation.

**Risk factors**	**Treated(*n* = 22)**	**Off treatment (*n* = 14)**	***P* value**
Age (years)	15.7 ± 5.4	17.1 ± 5.7	0.5
% fat by bioelectrical impedance	27.8 ± 6.8	23.0 ± 7.7	0.06
BMI percentile	61.0 ± 29.4	59.7 ± 23.9	0.8
SBP (mmHg)	113.7 ± 11.4	117.8 ± 7.4	0.2
SBP percentile	61.4 ± 27.7	73.7 ± 17.5	0.2
DBP (mmHg)	68.0 ± 8.2	66.7 ± 6.3	0.6
DBP percentile	60.6 ± 25.9	54.8 ± 17.8	0.4
Iliac skinfold (mm)	16.1 ± 8.9	14.2 ± 7.0	0.5
Triceps skinfold (mm)	18.4 ± 8.7	14.1 ± 6.6	0.1
Scapular skinfold (mm)	13.4 ± 9.6	9.3 ± 6.0	0.2
Waist circumference (cm)	74.1 ± 13.2	67.4 ± 11.9	0.1
Waist circumference >50th percentile, *n* (%)	12 (54.5)	5 (35.7)	0.2
Hip circumference (cm)	85.7 ± 15.5	79.2 ± 18.1	0.28
Waist/hip ratio	0.88 ± 0.13	0.87 ± 0.09	0.8
Total cholesterol (mg/dl)	187.5 ± 48.8	191.4 ± 44.3	0.8
HDL (mg/dl)	59.9 ± 19.3	57.2 ± 16.5	0.6
LDL (mg/dl)	107.4 ± 33.2	106.5 ± 27.8	0.9
Triglycerides (mg/dl)	88.6 ± 31.8	118.3 ± 66.5	0.07
Fasting Glucose (mg/dl)	84.8 ± 14.3	82.4 ± 10.6	0.6
Fasting Insulin (micU/ml)	5.6 ± 6.1	3.9 ± 2.7	0.5
HOMA-IR	1.1 ± 1.2	0.8 ± 0.4	0.48

*Data are mean ±SD unless otherwise indicated*.

*Untreated patients who underwent extensive evaluation (n = 2) were excluded due to small sample size*.

*HDL, high-density lipoprotein; HOMA-IR, homeostatic model assessment-insulin resistance; LDL, low-density lipoprotein*.

There were no significant differences by sex in anthropometric, laboratory, and metabolic variables (data not shown) except for a higher body fat mass (expressed in percent) in female than male patients (26.9 ± 6.8 vs. 19.2 ± 8.9%, *p* = 0.016), which we assumed was physiologic (27).

At the time of evaluation, the mean age of the 56 patients referred for premature pubarche was 17 ± 6.3 years, and of those referred for other presentations, 17.3 ± 7.4 years (*p* = 0.8). There were also no significant differences between these subgroups in any of the anthropometric, laboratory, and metabolic variables (data not shown).

## Discussion

This study showed that adolescents diagnosed with NCCAH in childhood did not have an increased prevalence of obesity or overweight compared to the general population in our country. Furthermore, the rate of obesity was similar to the rate in their parents, and the rate of overweight was even lower than that in their parents.

These results are not in agreement with the US National Institutes of Health study wherein overall 35% of children with CAH were obese, with no differences between the classic and the non-classic groups (15). Others reported a higher than normal risk of obesity in children and adolescents with classical CAH (32). The higher BMI-SDS was associated with increased glucocorticoid dosage, parental obesity, and chronological age (32). A Swedish study comparing patients with 21-hydroxylase deficiency, presenting as either salt-wasting, simple virilizing, or NCCAH, to national population registers found that obesity was most pronounced in the patients with NCCAH, who also had increased cardiovascular morbidity (16). However, data on age at diagnosis and glucocorticoid duration and dose as well as other clinical details were missing from the national registers. The authors suggested that the mild phenotype of NCCAH was associated with delayed diagnosis and consequently prolonged androgen excess, which may precipitate cardiovascular morbidity.

In our study, BMI-SDS was not associated with either initial or current glucocorticoid dose. Similarly, in an earlier study of adolescent patients with CAH, there was no association of BMI-SDS with either glucocorticoid dosage or age (13). BMI-SDS at the last clinic visit was negatively correlated with the duration of glucocorticoid therapy. This finding may be explained by the regular follow-up of the treated patients, with tight surveillance of weight changes and diet, including dietary consultation that was provided as part of the clinic visits when indicated.

Patients still under treatment tended to have a higher body fat mass than off-treatment patients (with a similar male-to-female ratio in both groups), despite comparable BMI-SDS and weight-SDS. This finding could be due to the effect of glucocorticoid excess on body composition (33). Our data are in line with those of Nermoen et al. (34) who found higher fat mass in patients treated by steroids for classical CAH than controls despite a similar BMI-SDS. The higher fat mass in patients under treatment may contribute to insulin resistance. Indeed, insulin and HOMA-IR were higher in our treated group, but the difference was not statistically significant, and in both groups these values were all within the normal range.

The lower fat mass in the off-treatment patients may point to a waning of the glucocorticoid effect on body composition once therapy is discontinued; to the known effect of androgen on reducing body fat mass, as androstenedione was found to be higher in the off-treatment group; or both.

In our cohort the prevalence of systolic hypertension (12.2%) was higher than the 3.5% reported in children and adolescents (22), with no significant difference by treatment status. Data regarding blood pressure in NCCAH patients are scant and inconclusive: Finkielstain et al. found hypertension in 20% of the NCCAH patients (15), while no overt hypertension was found in a Polish study of 9 patients with NCCAH and 61 with classical CAH (35). Williams et al. (36) reported higher SBP values in 12 children with NCCAH than in controls, but they did not translate the numbers into percentiles adjusted for age, sex, and height, and did not report the rate of hypertension. By contrast, others noted that DBP-SDS was higher in a group of 9 adolescents with NCCAH than in healthy controls (37) and was associated with higher arterial intima-media thickness. The authors suggested that intermittent iatrogenic hypercortisolism may play an important role in the pathogenesis of artery alterations in CAH. In our study, a higher SBP percentile was associated with a higher drug dose in the first year of therapy, but not with the duration of therapy. This finding could reflect the severity of disease and possibly higher androgen levels. However, neither SBP nor DBP was associated with androgen levels at diagnosis, suggesting that the glucocorticoid therapy was responsible for the higher blood pressure, with a sustained effect probably due to residual visceral adiposity and/or insulin resistance. The role of glucocorticoids in systolic hypertension is supported by an earlier study comparing exercise performance between patients with CAH and patients receiving a similar dose of glucocorticoids for juvenile idiopathic arthritis (38). These results suggested that the treatment rather than the CAH itself was responsible for the enhanced SBP response and other abnormalities (38). Given that high DBP is a marker of cardiovascular risk (39), our finding of a normal DBP in both the treated and untreated patients with NCCAH is encouraging.

Other than systolic hypertension, patients were not at higher risk of hyperlipidemia, impaired glucose metabolism, higher body fat mass, or change in body fat distribution. The waist circumference and waist-to-hip ratio in the adolescent female patients with NCCAH were similar to values in the general population, as opposed to youth with classical CAH, who were found in previous studies to have increased adiposity and an increased waist-to-hip ratio (13, 38).

Most patients with NCCAH require therapy until completion of linear growth, at which point it can be discontinued. In our study, most of the currently untreated patients (27/38) had been previously treated with glucocorticoids. It remains unclear if the effects of past long-term steroid administration persist after therapy is discontinued. Longitudinal studies of these patients into adult life are needed.

The strength of the present study lies in the relatively long follow-up of a cohort attending a single tertiary medical center. Using this design, we were able to investigate a group of previously treated patients and compare them to patients still under treatment. This is also the first study to evaluate rates of overweight and obesity in pediatric patients with NCCAH compared to their parents. Parental measurements were taken at our institute to ensure accuracy, in contrast to studies based on self-reported data which is inherently biased because participants tend to under-report weight and over-report height (40). This comparison may also be superior to comparison to healthy controls, as the weight status of children has been shown to be associated with the parental status (41), possibly owing to genetic, environmental, and nutritional influences. Our data are further empowered by comparison to the local general population.

Limitations of the study include the relatively small size of the subgroup of patients who underwent the more extensive metabolic evaluation and the lack of a control group. The diagnosis was genetically confirmed in most but not all of the patients. Few data were available on inflammatory markers, considered to represent metabolic derangement. We did not evaluate other risk factors such as serum adiponectin, C-reactive protein, homocysteine levels, and arterial intima-media thickness, and we were unable to assess family history of cardiometabolic risk factors. Similar to previous reports (6, 7), there were more female than male patients in our study group, as male patients are often overlooked. Thus, although no gender difference was found in our cohort, we were cautious and chose not to present the results.

In summary, NCCAH diagnosed in childhood, whether treated or untreated, is not associated with an increased risk of overweight, obesity, or metabolic derangement except for a higher rate of systolic hypertension. Larger, longer-term studies are needed to confirm our results.

## Precis

Pediatric patients with NCCAH do not appear to be at increased risk of overweight, obesity, or metabolic derangement, regardless of treatment, compared to the same age group in the general population.

## Data Availability Statement

All datasets generated for this study are included in the manuscript/Supplementary Files.

## Ethics Statement

This study was carried out in accordance with the recommendations of Declaration of Helsinki. The protocol was approved by the ethical committee of RMC.

## Author Contributions

LV contributed to the conception and design of the study, acquisition and interpretation of the data, prepared the first draft, and revised the manuscript. YL contributed to the acquisition and interpretation of the data and reviewed the manuscript critically for important intellectual content. MP, SS, and AT contributed to the acquisition of the data and reviewed the manuscript critically for important intellectual content. RB contributed to the conception and design of the study, acquisition and interpretation of the data, and revised the manuscript. Each author listed on the manuscript has seen and approved the submission of this version of the manuscript and takes full responsibility for the manuscript.

### Conflict of Interest

The authors declare that the research was conducted in the absence of any commercial or financial relationships that could be construed as a potential conflict of interest.

## Abbreviations

ACTH: adrenocorticotropic hormone
BMI: body mass index
CAH: congenital adrenal hyperplasia
DBP: diastolic blood pressure
HDL: high-density lipoprotein
IDF: International Diabetes Federation
IR: insulin resistance
LDL: low density lipoprotein
NCCAH: non-classical congenital adrenal hyperplasia
17-OHP: 17-hydroxyprogesterone
SBP: systolic blood pressure
TC: total cholesterol
TG: triglycerides.
