# Supplementary material for: Obesity and Cardiometabolic Risk Factors in Children and Young Adults With Non-classical 21-Hydroxylase Deficiency
Source: Front Endocrinol (Lausanne). 2019 Oct 11;10:698. doi: 10.3389/fendo.2019.00698 (PMC6798148; doi:10.3389/fendo.2019.00698)
Supplement: Supplementary file 1 [file Table_1.docx]

| P value | Treated  (n=103) | Untreated  (n=11) | Characteristics |
| --- | --- | --- | --- |
| At diagnosis | | | |
| 0.8 | 7.9 ± 4.0 | 7.8 ± 5.6 | Age (yrs) |
| 0.001 | 0.36 ± 1.09 | -0.99 ± 1.93 | Weight-SDS |
| 0.001 | 0.24 ± 1.09 | -1.07 ± 1.6 | Height-SDS |
| 0.14 | 0.41 ± 1.04 | -0.22 ± 1.7 | BMI-SDS |
| 0.005 | 1.16 ± 1.24 | 0.00 ± 0.83 | Δ Bone age-chronological age (yrs) |
| <0.001 | 31.0 ± 28.3 | 10.5 ± 7.1 | Basal 17OHP (nmol/l) |
| 0.13 | 134.8 ± 100.1 | 88.1 ± 52.4 | Stimulated 17OHP (nmol/l) |
| <0.05 | 356 ± 139 | 270 ± 89 | Basal cortisol (nmol/l) |
| 0.22 | 486 ± 110 | 534 ± 137 | Stimulated Cortisol |
| <0.03 | 4.38±4.03 | 1.02±0.7 | Androstenedione (nmol/l) |
| 0.07 | 0.90±0.97 | 2.17±6.08 | Testosterone (nmol/l) |
| At most recent evaluation | | | |
| <0.003 | 17.7 ± 6.7 | 11.4 ± 5.6 | Age (yrs) |
| <0.002 | 0.06 ± 1.29 | -0.69 ± 1.75 | Weight-SDS |
| 0.6 | -0.42 ± 0.95 | -0.57 ± 1.76 | Height-SDS |
| 0.09 | 0.34 ± 1.00 | 0.65 ± 0.89 | BMI-SDS |

Supplementary Table 1. Clinical characteristics of patients with NCCAH who were never treated compared to the rest of the cohort

Data are mean ±SD unless otherwise specified.

SDS = standard deviation score; BMI = body mass index; SBP=systolic blood pressure; DBP=diastolic blood pressure; 17OHP=17-hydroxyprogesterone
